# Research trends, hotspots, and thematic evolution in scoliosis and osteoporosis: a bibliometric mapping review

**DOI:** 10.3389/fsurg.2026.1878578

**Published:** 2026-07-23

**Authors:** Chao Jia, Hui Yang, Tengyun Yang, Leijie Chen

**Affiliations:** 1 Department of Orthopedics, The Second Affiliated Hospital, Kunming Medical University, Kunming, China; 2 The Third Affiliated Hospital of Kunming Medical University, Yunnan Cancer Hospital, Kunming, China

**Keywords:** adult spinal deformity, bibliometric review, bone mineral density, osteoporosis, scoliosis

## Abstract

**Background:**

Research on scoliosis and osteoporosis has increased steadily, but the research landscape and thematic evolution of this field remain incompletely characterized. This study aimed to provide a bibliometric mapping review of publication trends, collaboration patterns, research hotspots, and emerging directions in this interdisciplinary area.

**Methods:**

A bibliometric mapping review of published literature was conducted using the Web of Science Core Collection (WOSCC) and Scopus. Relevant publications published between January 1, 2006, and December 31, 2025, were identified. Bibliometrix, CiteSpace, and VOSviewer were used to analyze collaboration networks, keyword co-occurrence, burst references, and thematic evolution.

**Results:**

A total of 1,289 records were identified, and 1,013 publications were included after deduplication, including 810 articles and 203 reviews. Annual publication output increased steadily from 2006 to 2025, with an R^2^ of 0.97 in the merged dataset, whereas mean total citations per article showed an overall downward trend. The United States and China were the leading contributing countries/regions, and the University of California was the most productive institution. *European Spine Journal* and *Spine* were the leading source journals. Keyword and thematic analyses highlighted adult spinal deformity, spine surgery, bone quality assessment, and mechanical complications as major recent topics.

**Conclusions:**

Research on scoliosis and osteoporosis has expanded substantially over the past 2 decades and has become an increasingly multidisciplinary field led primarily by the United States and China. The field has shifted toward bone quality assessment, risk stratification, complication prediction, and comprehensive management.

## 1 Introduction

Scoliosis is a common three-dimensional spinal deformity that may occur in adolescent idiopathic scoliosis, adult spinal deformity, degenerative scoliosis, and certain syndromic or neuromuscular disorders (1, 2). With population aging and continued advances in spine surgery, the clinical focus of scoliosis research has broadened from deformity morphology and corrective treatment to long-term outcomes, complication risk, and perioperative management (3). This shift is particularly relevant in adult spinal deformity and degenerative scoliosis, where osteoporosis and low bone mass may compromise vertebral strength and bone quality and thereby affect fixation stability, fusion outcomes, proximal junctional complications, and the risk of revision surgery (4–6).

Bone quality has also gained increasing attention across different scoliosis subgroups. In adolescent idiopathic scoliosis, prior studies and systematic reviews have shown that some patients have reduced bone mineral density or persistent low bone mass, and the relationship between these abnormalities and curve progression remains an area of interest (7, 8). In adult spinal deformity and degenerative scoliosis, the relationship between osteoporosis-related structural fragility and mechanical complications, perioperative risk, and long-term outcomes is more direct. Accordingly, studies addressing bone health optimization, bone density and bone quality assessment, Hounsfield unit measurements, and other imaging-based surrogate markers have increased substantially (4, 6, 9). Together, these developments suggest that the intersection of scoliosis and osteoporosis has emerged as a clinically meaningful and increasingly distinct research area (10).

Despite this growing interest, the literature remains fragmented across multiple clinical subgroups, including adolescent idiopathic scoliosis, adult spinal deformity, degenerative scoliosis, neuromuscular disorders, and genetically related spinal deformities. In addition, the major contributors, international collaboration patterns, core journals, knowledge bases, and thematic evolution of this field have not been comprehensively characterized. Previous bibliometric studies have focused mainly on scoliosis overall or on selected subtypes, including adolescent idiopathic scoliosis, adult degenerative scoliosis, and early-onset scoliosis, whereas the research interface between scoliosis and osteoporosis remains insufficiently explored (11–13). A bibliometric mapping review is therefore well suited to characterize the broader knowledge landscape and emerging directions of this field (14).

Accordingly, this study integrated data from the Web of Science Core Collection (WOSCC) and Scopus to conduct a bibliometric mapping review of publications on scoliosis and osteoporosis published between 2006 and 2025, with particular emphasis on publication trends, collaboration patterns, core journals, research hotspots, knowledge bases, and thematic evolution.

## 2 Methods

### 2.1 Study design

This study was designed as a bibliometric mapping review of published literature on scoliosis and osteoporosis. The review aimed to characterize publication trends, collaboration patterns, knowledge structures, and thematic evolution in this field by integrating bibliometric methods with cross-database comparison. No prior protocol registration was performed.

### 2.2 Data sources and search strategy

A multi-database search strategy was used, with data obtained from the Web of Science Core Collection (WOSCC) and Scopus. Because WOSCC and Scopus have complementary strengths in disciplinary coverage and citation indexing, combining the 2 databases may broaden literature coverage and improve the robustness of bibliometric findings (15, 16). According to the study design, the WOSCC dataset, the Scopus dataset, and the merged WOSCC + Scopus dataset were used for overall feature comparison, trend validation, and knowledge structure analysis, respectively.

The literature search was completed on February 1, 2026, covering publications from January 1, 2006, to December 31, 2025. The search strategy was constructed using Boolean operators to combine search terms related to scoliosis and osteoporosis. The search query for WOSCC was TS = (scoliosis AND osteoporos*), whereas the search query for Scopus was TITLE-ABS-KEY(scoliosis) AND TITLE-ABS-KEY(osteoporos*). In Scopus, publication year was further restricted to 2006–2025, language was limited to English, and non-target document types were excluded. In both databases, only English-language publications were included, and document types were restricted to articles and reviews.

Data integration and deduplication in this study followed standardized procedures used in previous bibliometric studies (17, 18). Data integration and duplicate removal were performed using the bibliometrix package in R and custom Python scripts. Duplicate records were identified and excluded by comparing metadata, including article titles, authors, and publication years. A total of 276 duplicate records were removed. Finally, 1,013 unique publications were included in the analysis, comprising 810 original research articles and 203 review articles. The detailed search strategies for the WOSCC and Scopus databases are provided in Supplementary Table S1. The titles, abstracts, and keywords of all included publications were integrated to construct the corpus for subsequent analyses. During data preprocessing, data cleaning and standardization procedures implemented in the bibliometrix R package were applied to text fields, institutional names, and other bibliographic information. These procedures included case normalization, standardization of punctuation and symbol formats, and removal of redundant whitespace to reduce noise introduced by formatting inconsistencies.

**Figure 1:**
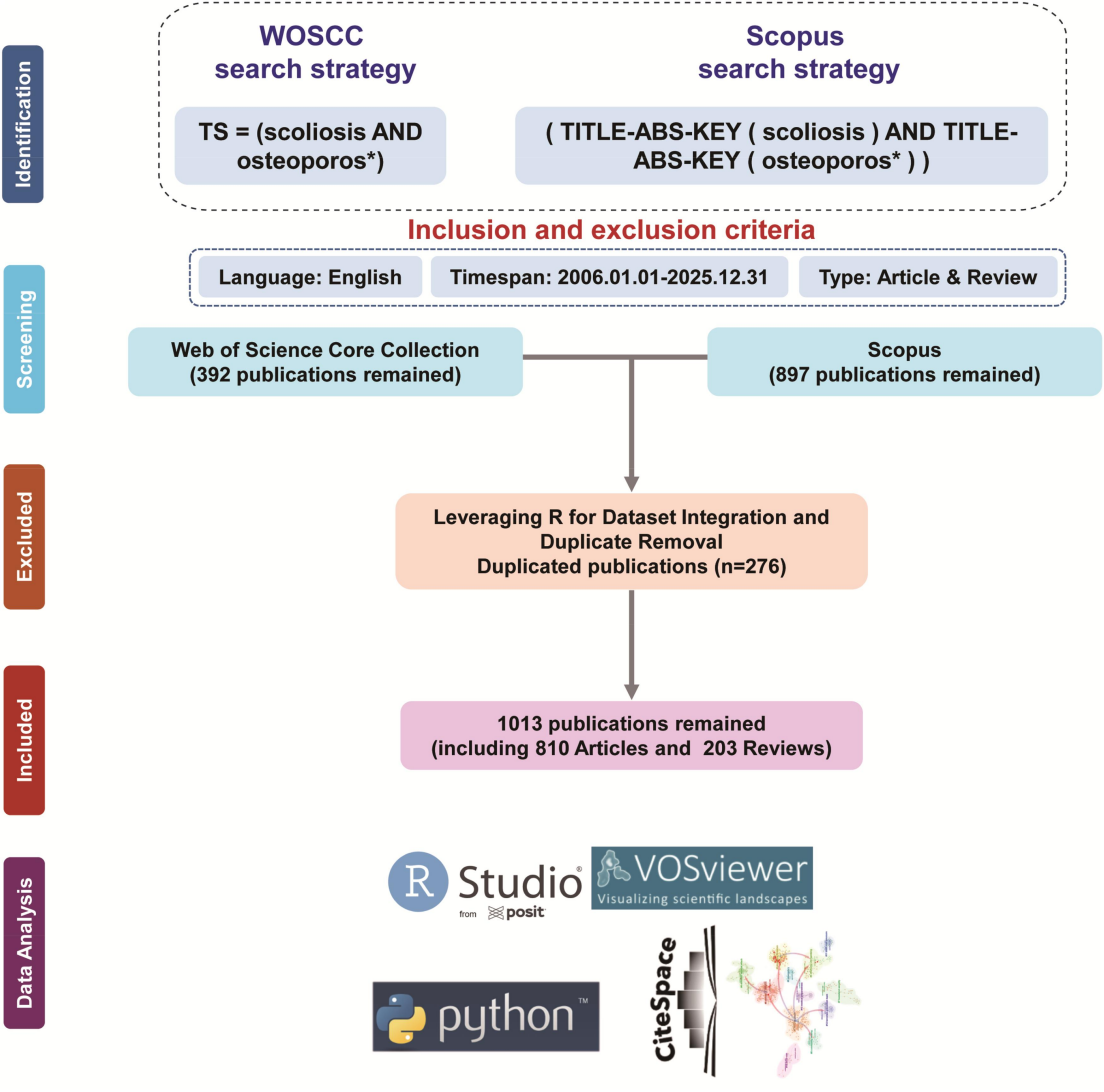
PRISMA-style flow diagram of study identification, screening, and inclusion.

The study selection process was summarized using a PRISMA-style flow diagram (Figure 1), in accordance with the PRISMA 2020 statement (19).

### 2.3 Bibliometric analysis

A multidimensional quantitative bibliometric analysis of the included publications was performed using multiple bibliometric tools. The bibliometrix package in R (R v4.4.5; bibliometrix v5.0.1) was used for basic descriptive analyses, including annual publication trends, citation counts, distributions of core countries, authors, and journals, and citation analyses. Raw network data were also extracted using this package (20).

A document co-citation network was constructed to identify the core knowledge structure of the field. The top 50 most frequently cited nodes were selected for analysis, and community detection was performed using the Louvain algorithm. To optimize the network topology, the repulsion force parameter was set to 0.5, and an automatic layout algorithm was applied. Isolated nodes and weakly connected nodes with fewer than two links were removed to ensure the connectivity and interpretability of the network map.

Trend Topics analysis was conducted using the bibliometrix package in R to identify temporal changes in frequently occurring research terms. The top 100 high-frequency terms were extracted, and thematic clustering was performed using the Louvain algorithm, with the minimum frequency threshold set at 5 occurrences per 1,000 publications. Two core labels were displayed for each thematic cluster to summarize the major research topics.

Thematic evolution analysis was performed using authors' keywords to trace the dynamic development of research themes over time. The top 250 high-frequency terms were selected, and core terms were screened using a minimum frequency threshold of 5 occurrences per 1,000 publications. For weighting, an inclusion index based on term-frequency weighting was used, and the minimum weight index threshold was set to 0.1. Network construction and community detection were conducted using the Louvain algorithm, with the repulsion force parameter set to 0.5. The final thematic evolution map incorporated a timeline to show the evolution of core themes across different periods. Only the top three labels with the highest weights in each cluster were displayed, with a label size coefficient of 0.3, to provide a clear visualization of the dynamic developmental trajectory of the research field.

Collaboration network analysis was performed using the bibliometrix package in R. First, a co-occurrence matrix was constructed using the biblioNetwork() function and normalized using the association strength method to reduce the influence of scale differences. For visualization, network maps were generated using the networkPlot() function. The number of nodes was limited to the top 30 most frequent nodes, and isolated nodes without links were removed. An automatic layout algorithm was used, and the repulsion force parameter was set to 0.5 to optimize the spatial distribution of the network. The Louvain algorithm was also applied for community detection to identify the major clusters within the collaboration network.

Multiple correspondence analysis (MCA) implemented in the bibliometrix package was used for dimensionality reduction of high-dimensional bibliographic data. The top 50 most frequent terms were selected as analytical units and grouped into three clusters to identify and visualize the major thematic clusters and their internal associations in this research field.

CiteSpace (v6.4.R1) was used for keyword clustering analysis. The time slicing interval was set to 1 year, and node selection followed the following parameters: g-index (*k* = 25), LRF = 2.5, L/N = 10, LBY = 5, and e = 1.0. Keyword burst detection and citation burst analysis in CiteSpace were performed with the minimum duration set to 2 years, while the remaining algorithmic parameters, including the state transition rate *α* and weight *γ*, were kept at their default values (*α* = 2.0 and *γ* = 1.0) (21). In addition, VOSviewer (v1.6.20) was used to construct a keyword co-occurrence clustering network, with the minimum keyword occurrence threshold set to 5 (20). Part of the raw data generated by the above software programs was imported into Python (v3.14) for further visualization using pandas, matplotlib, seaborn, and networkx.

### 2.4 Cross-database comparison and validation

**Figure 2:**
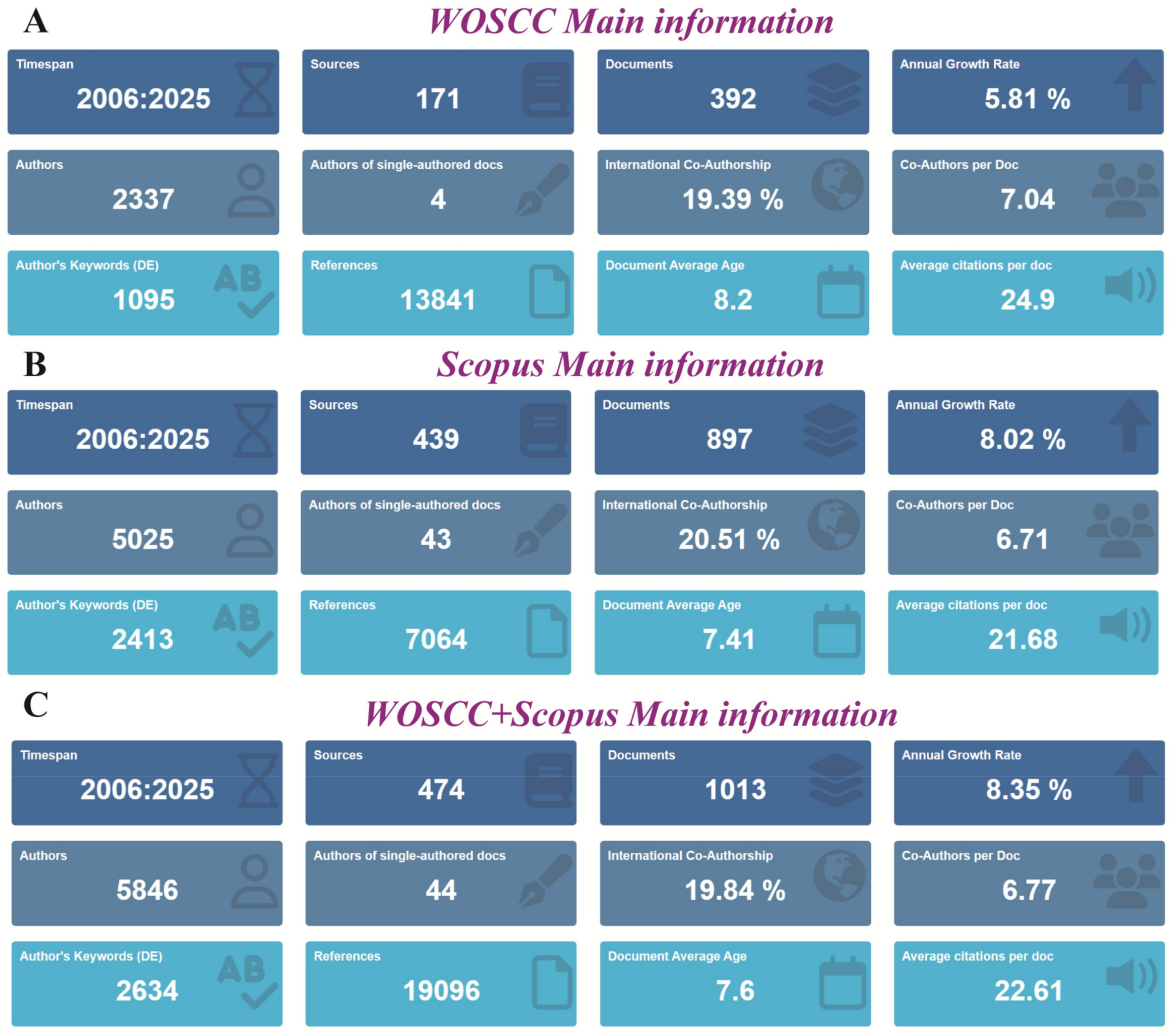
Comparison of the overall bibliometric characteristics across datasets. **(A)** Overall bibliometric characteristics of the WOSCC dataset. **(B)** Overall bibliometric characteristics of the Scopus dataset. **(C)** Overall bibliometric characteristics of the merged WOSCC + Scopus dataset.

To address differences in database coverage, journal inclusion, standardization, and citation structure, the WOSCC, Scopus, and merged datasets were compared and cross-validated. The WOSCC and Scopus datasets were used to validate annual publication trends, collaboration patterns, and major country/region distributions, whereas the merged WOSCC + Scopus dataset was used for the main analyses of knowledge structure, keyword co-occurrence, and thematic evolution. Comparisons among the 3 datasets are shown in Figure 2 and Table 1.

**Table 1:** Comparison of overall bibliometric characteristics across the WOSCC, scopus, and merged WOSCC + scopus datasets.

Description	WOSCC	Scopus	WOSCC + Scopus
Timespan	2006:2025	2006:2025	2006:2025
Sources (Journals, Books, etc)	171	439	474
Documents	392	897	1,013
Annual Growth Rate %	5.81	8.02	8.35
Document Average Age	8.2	7.41	7.6
Average citations per doc	24.9	21.68	22.61
References	13,841	7,064	19,096
Keywords Plus (ID)	1,216	8,313	8,291
Author's Keywords (DE)	1,095	2,413	2,634
Authors	2,337	5,025	5,846
Authors of single-authored docs	4	43	44
Single-authored docs	4	47	47
Co-Authors per Doc	7.04	6.71	6.77
International co-authorships %	19.39	20.51	19.84
article	328	707	810
review	64	190	203

## 3 Results

### 3.1 Study selection and bibliographic characteristics

According to the search strategy, a total of 1,289 publications were retrieved from WOSCC and Scopus, including 392 from WOSCC and 897 from Scopus. After dataset merging and deduplication, 1,013 publications were included in the analysis, comprising 810 articles and 203 reviews (Figure 1).

The included publications were published between 2006 and 2025 and comprised 474 source journals, 5,846 authors, 19,096 cited references, and 2,634 Author's Keywords. The annual growth rates of the WOSCC and Scopus datasets were 5.81% and 8.02%, respectively, whereas the mean citations per document were 24.90 and 21.68, respectively (Table 1; Figures 2A,B). In the merged dataset, the annual growth rate was 8.35%, the mean document age was 7.6 years, the average number of citations per document was 22.61, the average number of authors per document was 6.77, and the proportion of internationally collaborative publications was 19.84% (Table 1; Figure 2C).

### 3.2 Trends in annual publication output and citation impact

**Figure 3:**
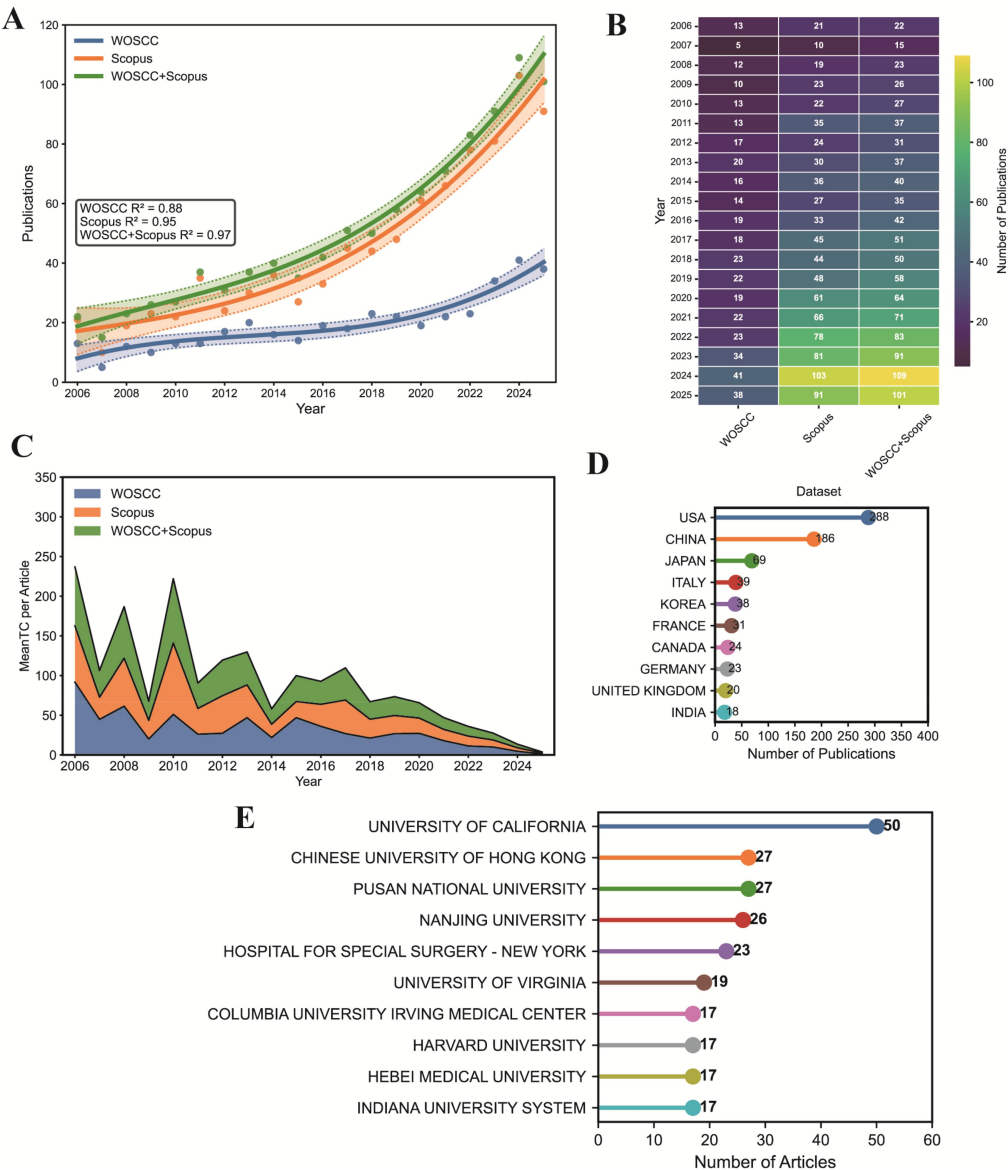
Publication trends and geographic distribution. **(A)** Annual publication trends and fitted curves in the WOSCC, Scopus, and merged WOSCC + Scopus datasets. **(B)** Quantitative comparison of annual publication output across the WOSCC, Scopus, and merged WOSCC + Scopus datasets. **(C)** Annual citation analysis across the WOSCC, Scopus, and merged WOSCC + Scopus datasets. **(D)** Lollipop plot of the top 10 most productive countries/regions in the merged WOSCC + Scopus dataset. **(E)** Lollipop plot of the top 10 most productive institutions in the merged WOSCC + Scopus dataset.

From 2006 to 2025, annual publication output showed a clear upward trend (Figures 3A,B). In the merged dataset, the number of publications increased from 22 in 2006 to 109 in 2024, with 101 publications in 2025. Curve fitting showed that the coefficients of determination (R²) for the WOSCC, Scopus, and merged datasets were 0.88, 0.95, and 0.97, respectively (Figure 3A).

In contrast to the increasing publication output, mean total citations per article demonstrated an overall downward trend (Figure 3C; Supplementary Table S2). In the merged dataset, mean total citations per article peaked at 81.00 in 2010, decreased to 19.20 in 2020, and further declined to 4.45 in 2024 and 1.22 in 2025. This decline may partly reflect citation-window effects, as more recent publications had less time to accumulate citations.

### 3.3 Distribution of countries/regions and institutions

Country/region-level analysis showed that the United States was the most productive contributor, with 288 publications (28.4% of the total), followed by China with 186 publications (18.4%) and Japan with 69 publications (6.8%) (Table 2; Figure 3D). Italy, South Korea, and France ranked next.

**Table 2:** Top 10 most productive countries/regions and their collaboration rates in the merged WOSCC + scopus dataset.

Country	Articles	Articles %	SCP	MCP	MCP %
USA	288	28.4	228	60	20.8
CHINA	186	18.4	164	22	11.8
JAPAN	69	6.8	62	7	10.1
ITALY	39	3.8	28	11	28.2
KOREA	38	3.8	35	3	7.9
FRANCE	31	3.1	26	5	16.1
CANADA	24	2.4	9	15	62.5
GERMANY	23	2.3	16	7	30.4
UNITED KINGDOM	20	2	13	7	35
INDIA	18	1.8	14	4	22.2

At the institutional level, the University of California ranked first with 50 publications, followed by The Chinese University of Hong Kong and Pusan National University, each with 27 publications. Nanjing University contributed 26 publications, and Hospital for Special Surgery contributed 23 publications (Figure 3E).

In terms of international collaboration, Canada had the highest proportion of multiple-country publications (MCP%), at 62.5%, followed by the United Kingdom, Germany, and Italy (Table 2).

### 3.4 Author impact metrics

Author impact metrics are presented in Table 3. Lenke Lawrence G and Shaffrey Christopher I had the highest h-index values, both of 10. Shaffrey Christopher I had the highest total citation count, at 481, whereas Li Weishi had the highest m-index, at 1.0.

**Table 3:** Top 10 authors ranked by bibliometric indicators in the merged WOSCC + scopus dataset.

Author	h_index	g_index	m_index	TC	NP	PY_start
Lenke Lawrence G	10	16	0.667	280	17	2012
Shaffrey Christopher I	10	13	0.476	481	13	2006
Bess Shay	8	9	0.8	328	9	2017
Kim Han JO	8	9	0.571	381	9	2013
Lafage Renaud	8	15	0.667	264	15	2015
Lafage Virginie	8	10	0.8	331	10	2017
Li Weishi	7	10	1	346	10	2020
Qiu Yong	7	10	0.467	117	13	2012
Smith Justin S.	7	9	0.7	253	9	2017
Ames Christopher P.	6	7	0.6	229	7	2017

### 3.5 Journal distribution and dual-map overlay analysis

Analysis of source journals showed that European Spine Journal was the most productive journal, followed by Spine, Osteoporosis International, Spine Journal, and Global Spine Journal (Supplementary Figure S1).

In terms of journal influence, Spine ranked first in h-index, g-index, and total citations, with values of 25, 45, and 2,146, respectively. European Spine Journal ranked second, with an h-index of 21 and 1,788 total citations (Table 4).

**Table 4:** Top 10 journals ranked by bibliometric indicators in the merged WOSCC + scopus dataset.

Source	h_index	g_index	m_index	TC	NP	PY_start
Spine	25	45	1.19	2,146	45	2006
European Spine Journal	21	42	1	1,788	54	2006
Spine Journal	15	21	0.789	728	21	2008
Osteoporosis International	14	22	0.667	883	22	2006
Journal of Neurosurgery-Spine	10	14	0.588	584	14	2010
Bone	9	15	0.45	502	15	2007
Global Spine Journal	9	17	1.286	290	20	2020
Spine Deformity	8	12	0.571	149	15	2013
American Journal of Medical Genetics Part A	7	7	0.438	265	7	2011
Journal of Pineal Research	7	7	0.368	564	7	2008

**Figure 4:**
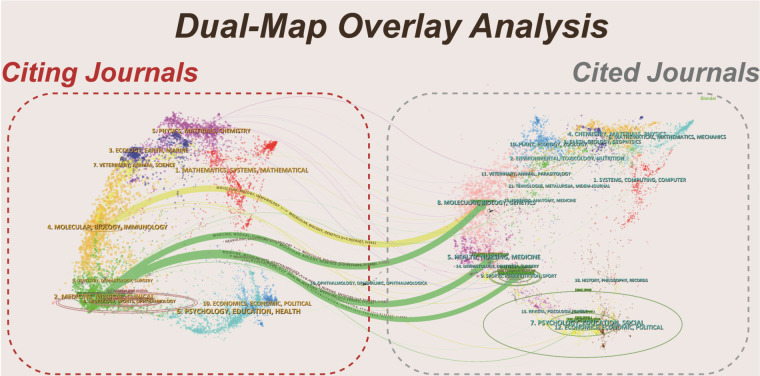
Dual-map overlay analysis of the merged WOSCC + scopus dataset.

Dual-map overlay analysis showed that the citing journals were mainly distributed in Medicine, Medical, Clinical and Molecular, Biology, Immunology, whereas the cited journals were mainly distributed in Health, Nursing, Medicine and Molecular, Biology, Genetics (Figure 4).

### 3.6 Keyword co-occurrence, clustering, and trend topics

High-frequency keyword analysis showed that the most common keywords were osteoporosis, scoliosis, bone density, clinical article, follow up, and case report (Supplementary Table S3; Supplementary Figure S2).

**Figure 5:**
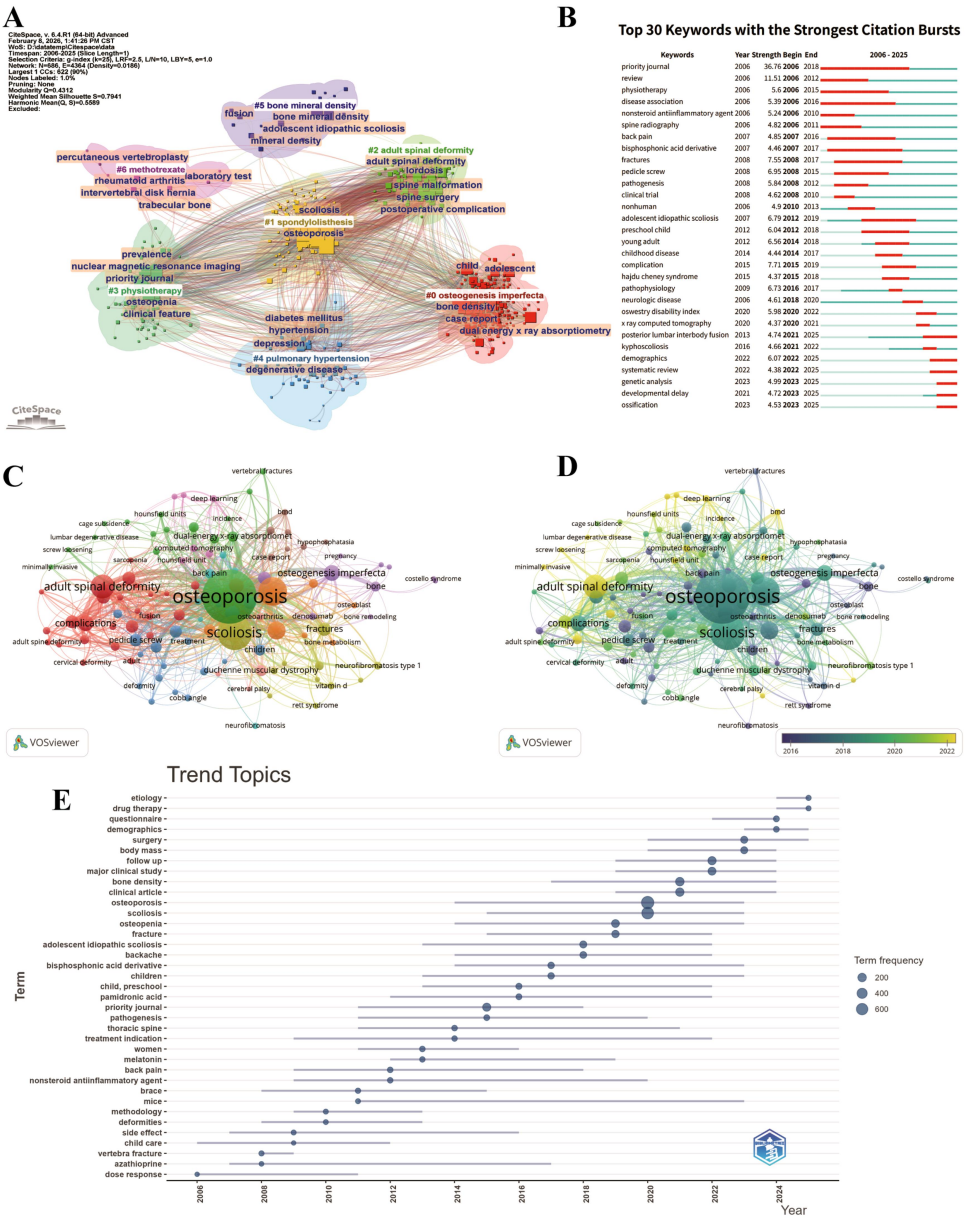
Keyword analysis. **(A)** Keyword clustering map of the merged WOSCC + Scopus dataset. **(B)** Top 30 keywords with the strongest citation bursts in the merged WOSCC + Scopus dataset. **(C)** Keyword co-occurrence clustering map of the merged WOSCC + Scopus dataset. **(D)** Temporal overlay visualization of keyword co-occurrence in the merged WOSCC + Scopus dataset. **(E)** Trend topics map of the merged WOSCC + Scopus dataset.

In the CiteSpace keyword clustering network, the modularity Q value was 0.4312 and the weighted mean silhouette S value was 0.7941, indicating acceptable clustering quality (Figure 5A). Seven major clusters were identified, including osteogenesis imperfecta, spondylolisthesis, adult spinal deformity, physiotherapy, pulmonary hypertension, bone mineral density, and methotrexate.

Burst keyword analysis showed that recent active themes included kyphoscoliosis, demographics, systematic review, genetic analysis, developmental delay, and ossification (Figure 5B).

In the VOSviewer keyword co-occurrence map, osteoporosis and scoliosis occupied central positions and were closely linked to adult spinal deformity, fractures, bone mineral density, and dual-energy x-ray absorptiometry (Figure 5C). The temporal overlay visualization further indicated increasing recent attention to adult spinal deformity, spine surgery, and bone density-related topics (Figure 5D).

Trend topics analysis indicated increasing attention to spine surgery, adult spinal deformity, scoliosis, osteoporosis, and bone density in recent years (Figure 5E).

### 3.7 Collaboration networks and productivity patterns

**Figure 6:**
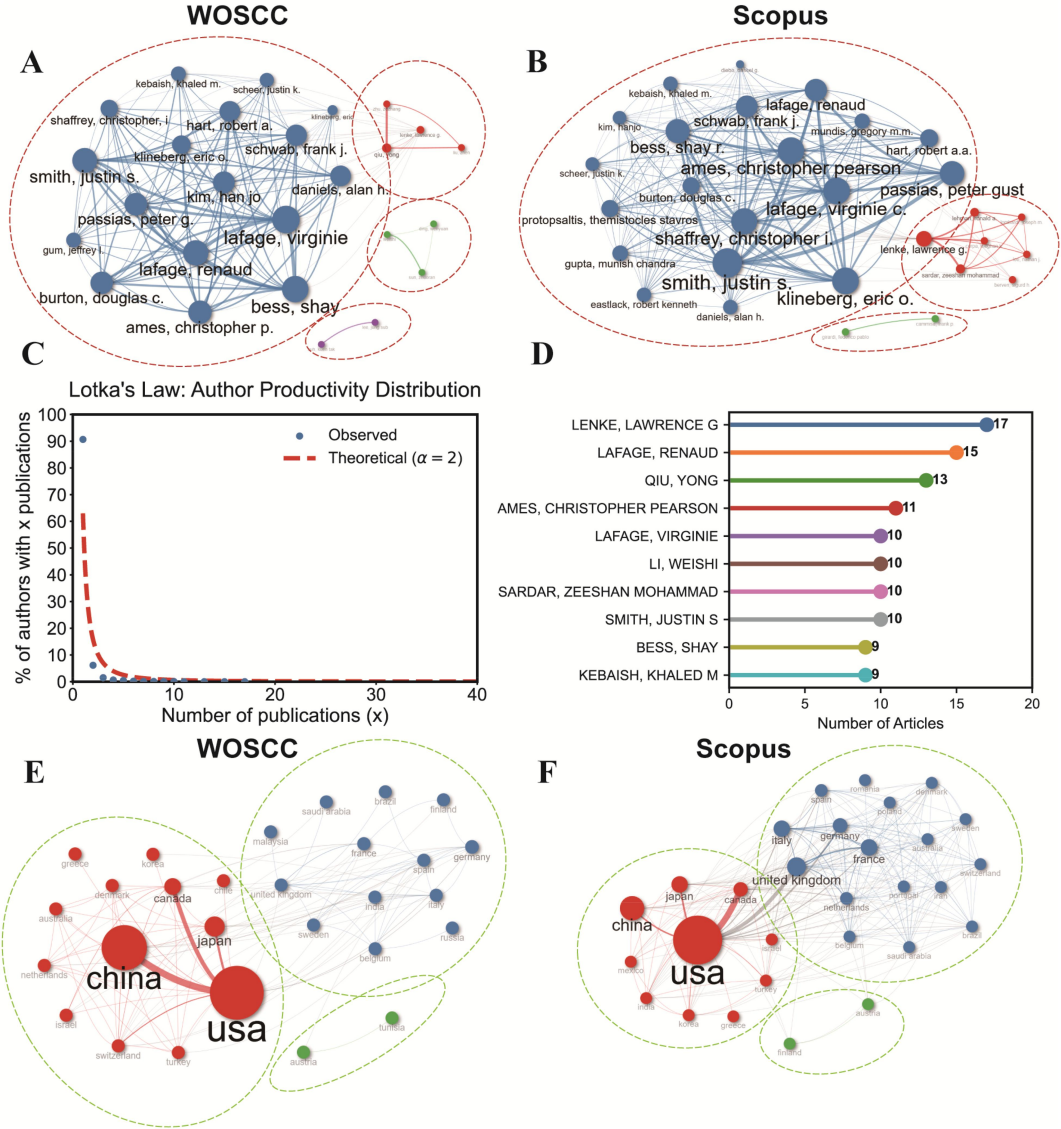
Comparative validation analysis of collaboration patterns among authors and countries/regions. **(A,B)** Author collaboration networks in WOSCC and Scopus. **(C)** Author productivity distribution in the merged WOSCC + Scopus dataset. **(D)** Top 10 most productive authors in the merged WOSCC + Scopus dataset. **(E,F)** Country/region collaboration networks in WOSCC and Scopus.

The author collaboration network showed that Lafage Virginie, Lafage Renaud, Smith Justin S, Klineberg Eric O, Ames Christopher P, and Shaffrey Christopher I formed the major collaborative cluster in this field (Figures 6A,B). The Lotka's law distribution indicated that most authors published only 1 article, and the number of authors decreased progressively as publication count increased (Figure 6C).

Among the most productive authors, Lenke Lawrence G ranked first with 17 publications, followed by Lafage Renaud with 15 and Qiu Yong with 13 (Figure 6D). Ames Christopher *P* published 11 articles, whereas Lafage Virginie, Li Weishi, Sardar Zeeshan Mohammad, and Smith Justin S each published 10.

The country collaboration network showed that the United States and China occupied prominent positions with relatively strong collaborative links, while a moderate collaboration network was also observed among European countries (Figures 6E,F).

### 3.8 Highly cited publications and burst reference analysis

**Figure 7:**
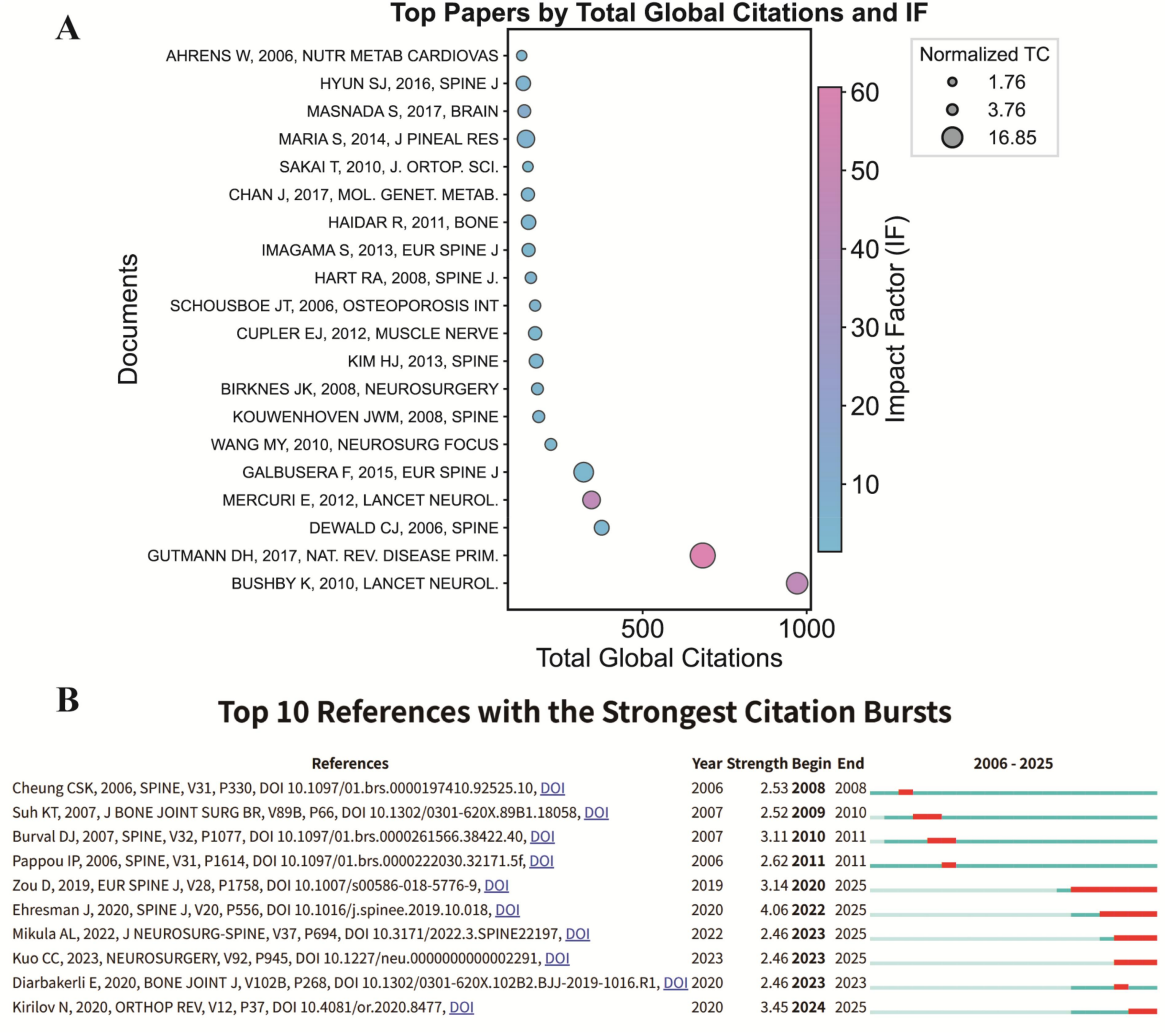
Citation analysis. **(A)** Bubble plot of the top 20 publications by total global citations, journal impact factor, and normalized total citations in the merged WOSCC + Scopus dataset. **(B)** Top 10 references with the strongest citation bursts in the merged WOSCC + Scopus dataset.

The publication with the highest total global citations was the article by Bushby K, published in Lancet Neurology in 2010, with 971 total citations. The article by Gutmann DH, published in Nature Reviews Disease Primers in 2017, had 683 total citations but ranked first in both average citations per year (68.30) and normalized total citations (16.85). Other highly cited publications included Dewald CJ, Mercuri E, and Galbusera F (Table 5; Figure 7A).

**Table 5:** Top 20 globally cited publications and their detailed bibliometric characteristics in the merged WOSCC + scopus dataset.

Paper	DOI	Global Total Citations	TC per Year	Normalized TC
Bushby K, 2010, LANCET NEUROL.	10.1016/S1474-4422 (09)70272-8	971	57.12	11.99
Gutmann DH, 2017, NAT. REV. DISEASE PRIM.	10.1038/nrdp.2017.4	683	68.30	16.85
Dewald CJ, 2006, SPINE	10.1097/01.brs.0000236893.65878.39	375	17.86	5.05
Mercuri E, 2012, LANCET NEUROL.	10.1016/S1474-4422 (12)70061-3	344	22.93	7.67
Galbusera F, 2015, EUR SPINE J	10.1007/s00586-015-3768-6	320	26.67	9.82
Wang MY, 2010, NEUROSURG FOCUS	10.3171/2010.1.FOCUS09286	220	12.94	2.72
Kouwenhoven JWM, 2008, SPINE	10.1097/BRS.0b013e3181891751	183	9.63	2.82
Birknes JK, 2008, NEUROSURGERY	10.1227/01.NEU.0000325485.49323.B2	179	9.42	2.76
Kim HJ, 2013, SPINE	10.1097/BRS.0b013e3182815b42	175	12.50	4.22
Schousboe JT, 2006, OSTEOPOROSIS INT	10.1007/s00198-005-2010-5	172	8.19	2.32
Cupler EJ, 2012, MUSCLE NERVE	10.1002/mus.22329	172	11.47	3.83
Hart RA, 2008, SPINE J.	10.1016/j.spinee.2008.01.015	159	8.37	2.45
Imagama S, 2013, EUR SPINE J	10.1007/s00586-013-2721-9	152	10.86	3.66
Haidar R, 2011, BONE	10.1016/j.bone.2010.10.173	152	9.50	4.75
Chan J, 2017, MOL. GENET. METAB.	10.1016/j.ymgme.2016.12.004	150	15.00	3.70
Sakai T, 2010, J. ORTOP. SCI.	10.1007/s00776-010-1454-4	150	8.82	1.85
Maria S, 2014, J PINEAL RES	10.1111/jpi.12116	144	11.08	7.45
Masnada S, 2017, BRAIN	10.1093/brain/awx184	139	13.90	3.43
Hyun SJ, 2016, SPINE J	10.1016/j.spinee.2016.05.008	136	12.36	4.71
Ahrens W, 2006, NUTR METAB CARDIOVAS	10.1016/j.numecd.2006.01.011	131	6.24	1.76

Among the top 10 references with the strongest citation bursts, early burst references were mainly published in 2006–2007, whereas sustained recent bursts were largely concentrated in publications from 2019 onward (Figure 7B). The publication by Ehrensman J in 2020 showed the strongest burst (strength = 4.06), with the burst period spanning 2022–2025. Publications by Zou D, Mikula AL, Kuo CC, and Kirilov N also showed sustained citation bursts in recent years. Co-citation network analysis further characterized the intellectual structure of the field and identified several major knowledge clusters within the scoliosis–osteoporosis research interface (Supplementary Figure S4).

### 3.9 Thematic structure and evolution

**Figure 8:**
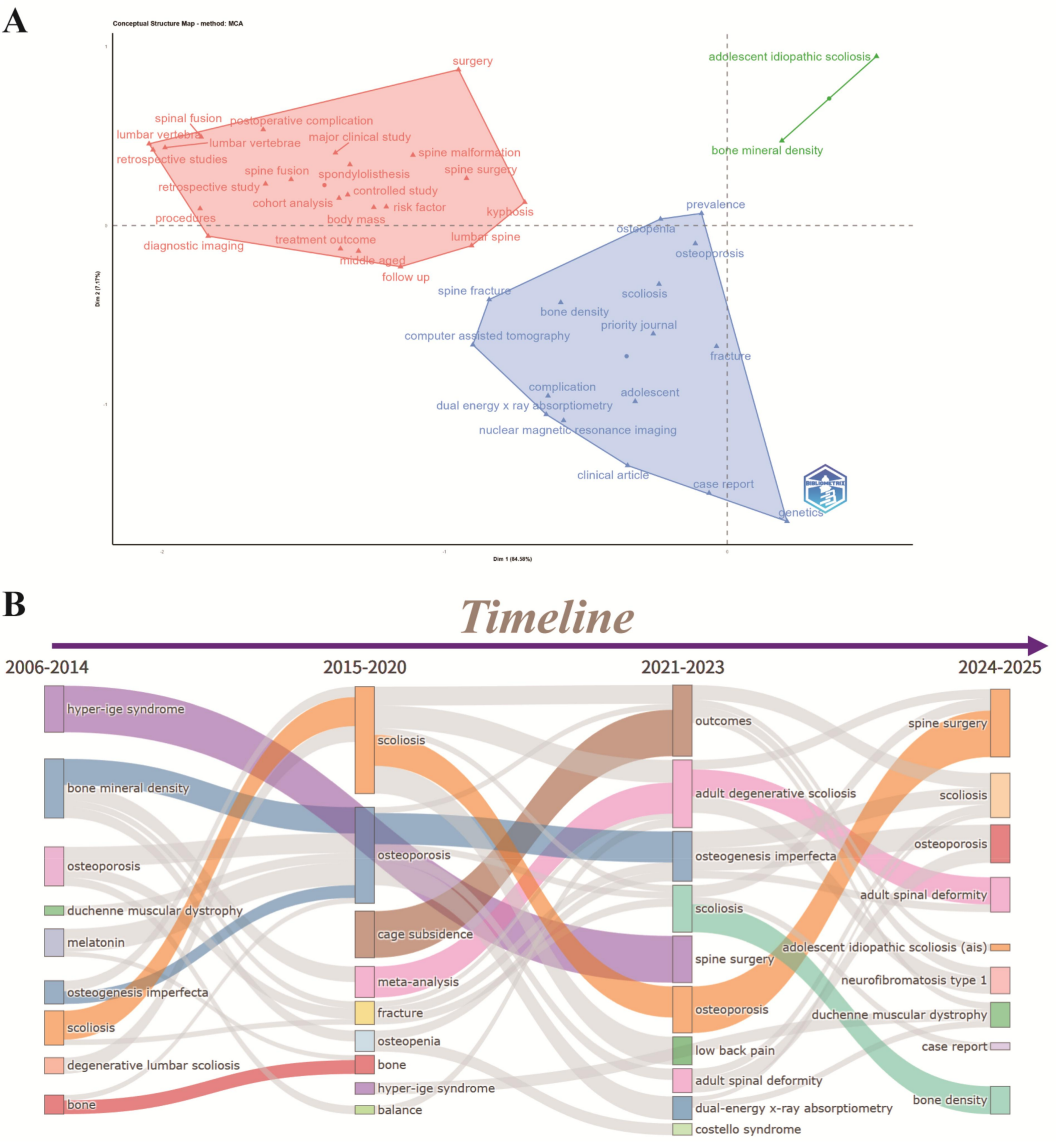
Analysis of thematic evolution trends. **(A)** Conceptual structure map based on multiple correspondence analysis (MCA) of the merged WOSCC + Scopus dataset. **(B)** Thematic evolution map of the merged WOSCC + Scopus dataset.

The conceptual structure map identified 3 major thematic groups in this field, broadly related to surgery, osteoporosis and imaging, and adolescent idiopathic scoliosis with bone mineral density (Figure 8A).

The thematic evolution analysis indicated increasing attention to spine surgery, adult spinal deformity, scoliosis, osteoporosis, and bone density over time (Figure 8B).

The thematic map showed that osteoporosis, bone mineral density, adolescent idiopathic scoliosis, and osteopenia were located in the motor themes quadrant, whereas.

scoliosis, spinal deformity, surgery, and lumbar spine were positioned in the basic themes quadrant (Supplementary Figure S3).

## 4 Discussion

Using an integrated WOSCC and Scopus dataset, this bibliometric mapping review provides an overview of research on scoliosis and osteoporosis published between 2006 and 2025. The field has expanded steadily over the past 2 decades, with progressively clearer research structure, shifting hotspot themes, and increasingly evident interdisciplinary characteristics. Overall, the literature appears to have evolved from early studies focused on disease association and imaging observations toward bone quality assessment, risk stratification, complication prediction, and comprehensive management.

### 4.1 Sustained growth in academic attention

Our analysis showed that publication output increased steadily from 2006 to 2025, with more pronounced growth after 2020. This pattern suggests that the intersection between scoliosis and osteoporosis has attracted increasing scholarly attention and has developed into a relatively stable area of research activity.

Several factors may have contributed to this growth. First, with population aging, the number of patients with adult spinal deformity and degenerative scoliosis has continued to increase, and low bone mass and osteoporosis are common in middle-aged and older populations with spinal disorders. Bone quality has therefore become an increasingly important consideration in the evaluation and treatment of spinal deformity (3). Second, as greater emphasis has been placed on long-term outcomes, the focus of spinal surgery research has gradually expanded from deformity correction alone to risk identification, outcome optimization, and long-term management (22–24). Third, with the accumulation of clinical evidence, the co-occurrence of osteoporosis and certain scoliosis phenotypes in older adults has received increasing attention and may be associated with a higher risk of spinal fragility fractures, further stimulating research interest in this field (25).

Although annual publication output has continued to increase, mean total citations per article showed an overall decline over time. This trend should be interpreted with caution, because citation performance in bibliometric studies is strongly influenced by time since publication, and recent publications have had less time to accumulate citations than earlier ones. Accordingly, the lower mean citation values observed in recent years should be interpreted primarily as citation-window bias rather than evidence of declining research quality or scholarly influence. Taken together with the sustained increase in publication output and the increasing refinement of research themes, these findings suggest that the field remains in an active phase of expansion and structural development.

### 4.2 The United States and China as major research hubs

Country/region-level analysis showed that the United States and China were the 2 major contributors to this field, with the United States occupying a particularly central position in terms of publication output, collaboration intensity, and network prominence. This pattern may be related to its long-standing strengths in adult spinal deformity research, multicenter clinical studies, spine registries, and longitudinal outcome investigations. For example, the American Spine Registry has integrated data from numerous spine centers and provides an important platform for evaluating surgical trends, effectiveness, and safety (26). China ranked second in publication output and has shown substantial growth in recent years, reflecting increasing research attention to osteoporosis-related topics (27). By contrast, although Canada, the United Kingdom, and Germany produced fewer publications overall, their higher proportions of multiple-country publications suggest that they may play a more prominent bridging role in international collaborative research.

At the same time, the author and country/region collaboration networks suggest that this field has not yet developed into a highly integrated global collaborative system. Existing collaborations are still concentrated within several relatively stable clusters, and intercluster or cross-regional collaboration remains limited. This pattern indicates that the field is still largely driven by a small number of high-impact centers and established research teams. To further improve evidence quality and the generalizability of findings, broader multicenter, multiregional, and standardized collaborative studies will be needed. This finding suggests that future studies should not only expand international collaboration, but also improve standardization in study definitions, imaging assessment, and outcome reporting.

### 4.3 Ongoing shifts toward bone quality and risk stratification

The high-frequency keywords suggest that the literature is centered on the intersection between spinal deformity and abnormalities in bone mass or bone quality rather than on any single clinical subtype. Because the keyword analysis was based on integrated keyword- and index-term-related fields exported from the databases, it is better interpreted as reflecting the overall knowledge structure of the field rather than author-provided keywords alone.

Keyword clustering showed that research has moved beyond asking whether scoliosis and osteoporosis are associated and has progressed toward more specific questions regarding how reduced bone quality influences spinal stability, treatment selection, and clinical outcomes (28).

Burst keyword analysis further suggests that current frontiers are concentrated in at least 3 areas: skeletal fragility and structural instability in scoliosis, particularly in adults (25); the potential value of imaging-based and quantitative approaches for bone quality assessment (29, 30); and more comprehensive evaluation of frailty, functional status, and long-term outcomes (31, 32).

Taken together, these findings suggest that the field is shifting from a disease-association framework toward one centered on bone quality and risk stratification. Importantly, this transition reflects changes in research priorities rather than evidence that any single assessment tool or management strategy has become an established clinical standard.

### 4.4 Interdisciplinarity as a defining feature of the field

Dual-map overlay analysis indicates that research on scoliosis and osteoporosis is inherently interdisciplinary, extending beyond spine surgery into broader biomedical and molecular domains (33, 34).

Although spine journals remain the main platforms for knowledge dissemination, journals focused on osteoporosis, bone biology, and related fields also contribute substantially (27, 35). Together with the prominence of imaging-, genetics-, and rehabilitation-related terms, this pattern suggests that the knowledge structure of the field is becoming increasingly multidimensional (36, 37).

For patients with scoliosis, particularly those with adult spinal deformity, deformity angle and alignment parameters alone may no longer be sufficient to explain overall clinical risk. Bone strength, bone quality, vertebral structure, muscle status, physiologic reserve, and postoperative recovery potential may all influence treatment strategy and prognosis (38, 39). Future development in the field is therefore likely to involve deeper integration of spine surgery, osteoporosis management, imaging assessment, rehabilitation medicine, and geriatrics (23, 40). This interdisciplinary shift also highlights the growing methodological importance of imaging and artificial intelligence in this field. Imaging-derived indicators, such as CT-based bone quality measures, may help refine risk stratification beyond conventional bone mineral density alone. Meanwhile, artificial intelligence and machine-learning approaches may support the integration of spinal alignment, bone quality, frailty, and other clinical variables for individualized complication prediction and surgical decision-making (41). However, these approaches still require external validation across different populations and clinical settings before they can be interpreted as robust decision-support tools.

### 4.5 Highly cited and burst references reveal both the knowledge base and the shift in current frontiers

Highly cited publications suggest that the knowledge base of this field is somewhat heterogeneous. Its core foundation is rooted in research on spinal deformity, adult scoliosis, structural imbalance, and treatment outcomes, but it also includes literature related to neuromuscular disorders, genetic syndromes, and secondary scoliosis (4, 42). This pattern is consistent with the presence of terms such as osteogenesis imperfecta in the keyword network and suggests that the search framework used in this study captures a field with relatively broad boundaries.

Broadly, the highly cited literature appears to fall into 2 groups. One consists mainly of publications in spine specialty journals and forms the key knowledge base related to deformity correction, adult scoliosis treatment, and mechanical complications (4, 43). The other includes studies on neuromuscular disease, genetic syndromes, and secondary scoliosis, indicating that within the framework of “scoliosis + osteoporosis”, the included literature is not restricted to adult degenerative scoliosis alone but also encompasses complex disorders characterized by both skeletal fragility and spinal deformity (42, 44). This broader composition should be considered when interpreting the clinical implications of the field. This broad scope was appropriate for mapping the overall scoliosis–osteoporosis research interface rather than a single clinical subgroup.

Burst reference analysis further showed that recent frontiers have shifted more clearly toward contemporary surgical outcomes, risk prediction, and newer forms of clinical evidence. Recent burst references have focused increasingly on mechanical complications, preoperative risk prediction, and structural assessment, suggesting that the field is moving from descriptive recognition toward risk management and decision support (4, 43).

### 4.6 Clinical implications

Although this bibliometric mapping review does not directly assess treatment efficacy, it can still inform future clinical research. The current hotspots and thematic evolution suggest that osteoporosis-related issues have become an important component of scoliosis research, particularly in adult spinal deformity and degenerative scoliosis (27, 36). The field is also moving from description of structural abnormalities toward risk identification, complication surveillance, and long-term outcome evaluation, indicating that bone density, imaging-based bone quality markers, functional status, and frailty may need to be incorporated more systematically into future study design and clinical assessment (45). More broadly, progress in this area will likely depend on closer collaboration across spine surgery, osteoporosis care, imaging, rehabilitation, and geriatrics.

### 4.7 Limitations

This study has several limitations. First, the search strategy relied primarily on the terms “scoliosis” and “osteoporos*”, which may have missed relevant studies focusing on bone quality, osteopenia, low bone mineral density, vertebral fragility, or imaging-based bone strength assessment if they did not explicitly use osteoporosis-related terminology. Therefore, the findings should be interpreted as reflecting the explicitly indexed scoliosis–osteoporosis literature rather than the entire body of research on scoliosis and bone quality. Second, only English-language publications were included, which may have led to underrepresentation of important studies published in other languages and introduced language bias. Third, although a combined WOSCC and Scopus retrieval strategy was used to improve literature coverage and the completeness of citation network analysis, the 2 databases still differ in coverage scope, citation linkage rules, author and institutional standardization, and keyword and index-term field settings, and such heterogeneity may have influenced some bibliometric results (46). Fourth, the included literature covered heterogeneous scoliosis-related conditions, including adolescent idiopathic scoliosis, adult spinal deformity, degenerative scoliosis, neuromuscular disorders, and genetically related skeletal disorders. Although this broad scope helped capture shared themes related to skeletal fragility, bone quality assessment, and multidisciplinary management, it limits the specificity of subgroup-level conclusions.

## 5 Conclusion

This study shows that research at the intersection of scoliosis and osteoporosis expanded steadily between 2006 and 2025 and has become an increasingly multidisciplinary field led primarily by the United States and China. The literature has shifted from early studies of disease association and imaging description toward bone quality assessment, complication risk identification, surgical outcome evaluation, and comprehensive management. Adult spinal deformity, degenerative scoliosis, quantitative assessment of bone quality, and multicenter standardized studies are likely to remain key directions for future research.

## Data Availability

The original contributions presented in the study are included in the article/Supplementary Material, further inquiries can be directed to the corresponding author/s.
